# ARDSFlag: an NLP/machine learning algorithm to visualize and detect high-probability ARDS admissions independent of provider recognition and billing codes

**DOI:** 10.1186/s12911-024-02573-5

**Published:** 2024-07-16

**Authors:** Amir Gandomi, Phil Wu, Daniel R Clement, Jinyan Xing, Rachel Aviv, Matthew Federbush, Zhiyong Yuan, Yajun Jing, Guangyao Wei, Negin Hajizadeh

**Affiliations:** 1 Frank G. Zarb School of Business, Hofstra University, Hempstead, NY USA; 2 Institute of Health System Science, Feinstein Institute for Medical Research, Manhasset, NY USA; 3 AiD Technologies, Stony Brook, NY USA; 4 Donald and Barbara Zucker School of Medicine at Hofstra/Northwell, Manhasset, NY USA; 5 Department of Critical Care Medicine, The Affiliated Hospital of Qingdao University, Qingdao, China; 6 Kaiser Permanente, Oakland, CA USA

**Keywords:** Acute respiratory distress syndrome (ARDS), Berlin criteria, Natural language processing (NLP), Machine learning, Large language models (LLM)

## Abstract

**Background:**

Despite the significance and prevalence of acute respiratory distress syndrome (ARDS), its detection remains highly variable and inconsistent. In this work, we aim to develop an algorithm (*ARDSFlag*) to automate the diagnosis of ARDS based on the Berlin definition. We also aim to develop a visualization tool that helps clinicians efficiently assess ARDS criteria.

**Methods:**

ARDSFlag applies machine learning (ML) and natural language processing (NLP) techniques to evaluate Berlin criteria by incorporating structured and unstructured data in an electronic health record (EHR) system. The study cohort includes 19,534 ICU admissions in the Medical Information Mart for Intensive Care III (MIMIC-III) database. The output is the ARDS diagnosis, onset time, and severity.

**Results:**

ARDSFlag includes separate text classifiers trained using large training sets to find evidence of bilateral infiltrates in radiology reports (accuracy of 91.9%±0.5%) and heart failure/fluid overload in radiology reports (accuracy 86.1%±0.5%) and echocardiogram notes (accuracy 98.4%±0.3%). A test set of 300 cases, which was blindly and independently labeled for ARDS by two groups of clinicians, shows that ARDSFlag generates an overall accuracy of 89.0% (specificity = 91.7%, recall = 80.3%, and precision = 75.0%) in detecting ARDS cases.

**Conclusion:**

To our best knowledge, this is the first study to focus on developing a method to automate the detection of ARDS. Some studies have developed and used other methods to answer other research questions. Expectedly, ARDSFlag generates a significantly higher performance in all accuracy measures compared to those methods.

**Supplementary Information:**

The online version contains supplementary material available at 10.1186/s12911-024-02573-5.

## Background

Acute respiratory distress syndrome (ARDS) is a rapidly progressive etiology of respiratory failure that is caused by inflammatory lung injury [1]. Damage to the cells that form a barrier around the alveoli (the small air sacs in the lung) causes them to fill with fluid, directly impeding normal gas exchange and leading to hypoxemia [2]. This process can be caused by a number of different conditions, including sepsis, trauma, pancreatitis, and smoke or corrosive chemical inhalation. ARDS is associated with a high mortality rate (~ 40%) and substantially impacts survivors’ quality of life [3, 4]. The definition of ARDS has evolved from the original definition in 1967 to the more recent 2012 Berlin criteria [5, 6]. The diagnosis of ARDS based on the Berlin definition requires a constellation of clinical findings, including bilateral pulmonary opacities on radiographic studies (not explained by lung collapse, pleural effusion, or lung masses) and no other etiology of alveolar fluid accumulation (i.e. cardiogenic edema or fluid overload). As a result, variability in the detection of ARDS remains problematic both in clinical practice and research [1, 7]– [10].

For example, the LUNG SAFE study, which was the largest multicenter cohort study of ARDS patients to investigate the epidemiology and outcomes of ARDS across 459 ICUs from 50 countries [8], found that, on average, 40% of ARDS patients identified by an automated algorithm using the Berlin criteria were not diagnosed by the clinicians. In addition, there was a delay in diagnosing ARDS among 66% of patients [8]. Early diagnosis of ARDS enables timely implementation of protective lung ventilation strategies and adjunctive measures [11], leading to lower mortality rates [8, 12, 13]. Furthermore, consistency in ARDS detection enables investigators to study the associations of treatment trajectories and patient characteristics with outcomes [14].

### Significance

This study contributes to the ARDS literature by developing *ARDSFlag*, a new method to automate the detection of ARDS based on structured and unstructured textual data stored in electronic health record (EHR) systems. ARDSFlag uses machine learning (ML) and natural language processing (NLP) techniques to evaluate Berlin criteria. ML and NLP have been proven to offer strong potential for identifying and predicting complex medical conditions by incorporating EHR data [15–17]. We also develop a visualization that integrates all components of the Berlin criteria in one graph. The use of this visualization may enhance the efficiency and accuracy of clinicians in detecting ARDS cases.

ARDSFlag evaluates the four parameters of the Berlin definition. It includes separate text classifiers trained using large training sets to detect bilateral infiltrates (BI) in radiology reports and heart failure/fluid overload (HF/FO) in radiology and echocardiogram (echo) reports. We use a validation set of 100 cases, developed by an independent review of two groups of clinicians, to find the optimal temporal sequence of Berlin parameters. Using a separate ground truth set of 300 cases, we show that the algorithm outperforms other methods in the literature, including the use of International Classification of Diseases (ICD) codes and the method developed by Serpa Neto et al. [18] It should be emphasized that the objective of cited studies is not to identify ARDS cases and our algorithm, to the best of our knowledge, the first one that focuses on this problem.

## Methods

### Dataset

**Fig. 1:**
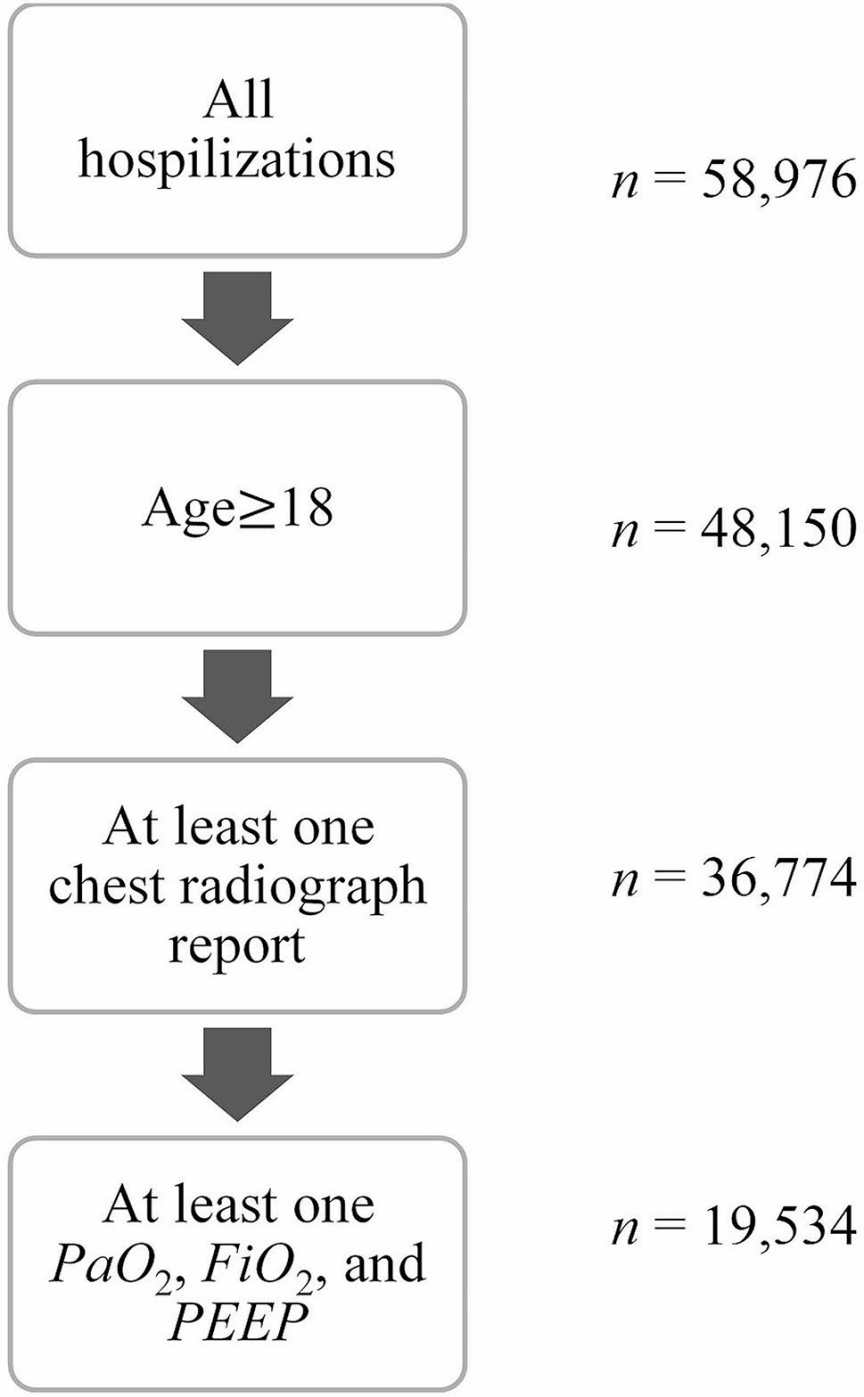
Cohort selection

We used the Medical Information Mart for Intensive Care III (MIMIC-III) dataset [19] to develop and test the automated ARDS detection algorithm. We used hospital admissions as the unit of analysis and, as shown in Fig. 1, limited the cohort to adult admissions (age$\ge$18). Since the Berlin definition is based on chest imaging reports, partial pressure of arterial oxygen (*PaO*_2_), fractional inspired oxygen (*FiO*_2_), and positive end expiratory pressure (*PEEP*), the cohort is further limited to admissions with at least one record of each. The inclusion criteria led to an initial cohort of 19,534 admissions.

Each patient’s relevant data points were fetched from MIMIC’s different tables and stored in individual multi-dimensional time series. We used Makhnevich et al.’s [20] algorithm to find the accurate intubation time. Another algorithm was developed to find the time of extubation based on recorded procedures, ventilator parameters, and oxygen delivery methods. Dispositions were mapped into four general categories *expired*, *hospice*, *home*, and *facility*, where the latter refers to locations such as skilled nursing facility, rehab, and short-/long-term hospital.

Central to the detection of ARDS is the evidence of bilateral infiltrates (BI) in chest radiographs and the diagnosis of cardiac failure or fluid overload ($HF/FO$). The NLP algorithms developed to extract such evidence from patient notes are described in a later subsection titled “NLP Algorithms.”

The data preprocessing pipeline was developed using Python 3.6. All factors related to ARDS detection are visualized in a single graph referred to as the *ARDS graph* hereafter. A sample ARDS graph is presented in Figure A1 in the Appendix. The corresponding time series is available in CSV format in supplementary files. To increase the efficiency of manual chart reviews, we developed a pipeline to print the structured admission data (e.g., demographics, admission and discharge dates, and the initial diagnosis), the ARDS graph, and all relevant notes for every case in a single PDF file. A set of keywords were selected to be highlighted in the pdf file.

### ARDS detection algorithm

#### Overview of the algorithm

Based on the Berlin criteria [21], ARDS is defined by: (1) acute onset, (2) *PaO*_2_*/FiO*_2_ (P/F ratio) ≤ 300 $mm Hg$ while Positive end-expiratory pressure (PEEP) ≥ 5 *cm H*_*2*_*O*, (3) BI in chest radiographs, and (4) the absence of HF/FO as the primary origin of pulmonary edema.

We used tracheostomy as a proxy to evaluate the first condition. If a patient had a tracheostomy within seven days of admission, they were classified as non-ARDS. Serpa Neto et al. [18] and Le et al. [9] have used the same proxy but with a 72-hour time window. Furthermore, the time of ARDS onset (determined based on the second and third criteria) must be within seven days after the first record of receiving$PEEP\ge 5$. For the second condition, we used the pre-processed $Pa{O}_{2}$, $Fi{O}_{2}$ and $PEEP$ recorded in the patient chart. For the third and fourth conditions, we trained different text classifiers. A text classifier was trained to find evidence of BI in chest radiology reports. Separate classifiers were developed to find $HF/FO$ in chest radiology and echo reports. Text classifiers are detailed in the next section.

**Fig. 2:**
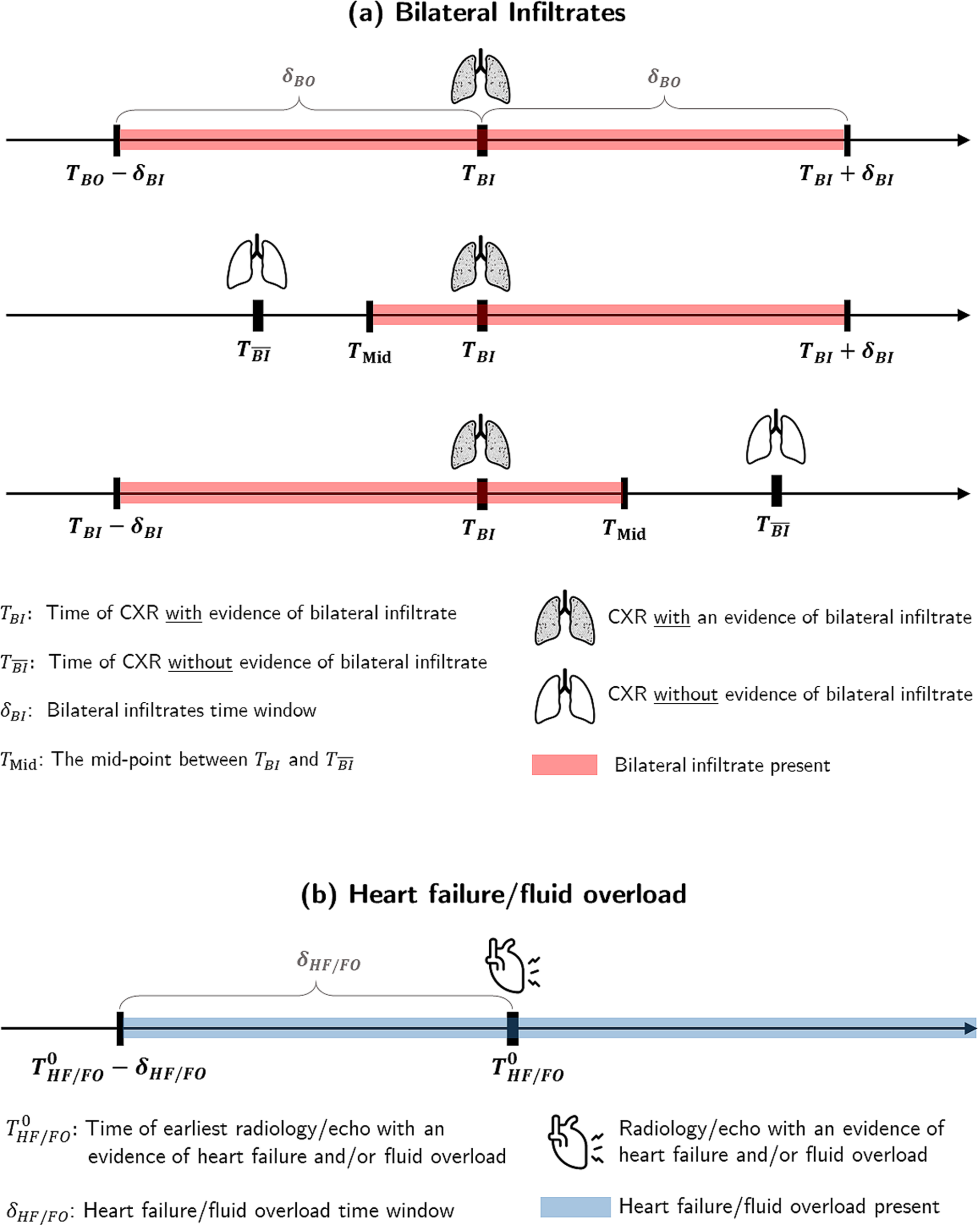
The timing of ARDS conditions: **a**. BI, **b**. Heart failure/fluid overload

Figure 2 depicts the logic for the sequence of conditions. As shown in the top graph in Fig. 2a, evidence of BI is generally valid within ${T}_{BI}\pm {\delta }_{BI}$, where ${T}_{BI}$ is the time of the radiology and ${\delta }_{BI}$ is the BI time window. A low $P/F$ ratio counts toward ARDS diagnosis if it occurs within this boundary. If there is another chest radiology *without* evidence of BI within ${T}_{BI}\pm {\delta }_{BI}$, as shown in the two bottom graphs in Fig. 2a, the boundary shrinks. As discussed later on, the optimal value of ${\delta }_{BI}$ is found to be one day.

Figure 2b shows the logic for the origin of edema. Let ${T}_{HF/FO}^{0}$ denote the time of the earliest echo/CXR with evidence of HF/FO, ${\delta }_{HF/FO}$ denote the corresponding time window, and ${T}_{0}$ denote the time of the potential ARDS onset (the earliest time the first three ARDS conditions are satisfied). If ${T}_{0}\ge {T}_{HF/FO}^{0}-{\delta }_{HF/FO}$ then HF/FO is identified as the origin of respiratory failure. Otherwise, ${T}_{0}$ is the time of ARDS onset. The optimal value of ${\delta }_{BI}$ will be shown to be five days. Figure A2 in Appendix further explains the Berlin implementation logic using a few sample cases.

To reduce the false-positive rate, we included the length of mechanical ventilation as an additional criterion; patients who received less than 48 h of mechanical ventilation were excluded unless they expired or were discharged to hospice within 48 h after intubation or extubation. Notably, we do *not* exclude short ventilations that are a result of severe illness (expire within 48 h after intubation) or elective extubation (expire or discharge to hospice within 48 h after extubation). Serpa Neto et al. [18] exclude patients receiving less than 48 h of ventilation regardless of whether the ventilation terminated due to death or elective palliative care, which may lead to the omission of severe cases. We perform a sensitivity analysis on this condition in the Discussion section.

#### Parameter tuning

We used a random set of 400 admissions to tune the algorithms’ parameters and evaluate their accuracy. In order to address the class imbalance issue, 100 of the 400 cases were randomly selected from a cohort that was classified as positive for ARDS by an initial version of the algorithm. The tuning parameters are ${\delta }_{BI}$ and ${\delta }_{HF/FO}$, the BI and HF/FO time windows. Two groups of clinicians were instructed to follow Berlin criteria and independently label cases for ARDS objectively. Each case’s relevant data was presented in a PDF file as previously described. Disagreements were settled by a joint evaluation (rate = 9%). We used 100 cases (25%) for parameter tuning and the remaining 300 cases as a test set to estimate the algorithm’s accuracy.

For the parameter tuning, we performed a grid search over values of ${\delta }_{BO}$ and ${\delta }_{HF/FO}$ in the set {4h, 8h, 12h, 1d, 2d, 5d, 7d} and used ${F}_{1}$-score to find the best combination of parameter values. ${\delta }_{BO}^{*}=1d$ and ${\delta }_{\text{H}\text{F}/\text{F}\text{O}}^{*}=5d$ generated the best result with ${F}_{1}=84\text{\%}$ ($\text{A}\text{c}\text{c}\text{u}\text{r}\text{a}\text{c}\text{y}=92\text{\%}$, $\text{P}\text{r}\text{e}\text{c}\text{i}\text{s}\text{i}\text{o}\text{n}=80.8\%$, $\text{R}\text{e}\text{c}\text{a}\text{l}\text{l}=87.5\text{\%}$). The optimal time windows (${\delta }_{BO}^{*}=1d$ and ${\delta }_{\text{H}\text{F}/\text{F}\text{O}}^{*}=5d$) are clinically relevant. ARDS is characterized by rapid onset of BI that can take weeks to months to fully resolve in most cases. Patients with fluid overload may initially meet Berlin criteria; however, the BI seen will usually improve rapidly with medical management. If a resolution of BI occurs within a matter of days, it is likely to result from a cardiogenic process or fluid overload as opposed to ARDS. Table 1 shows the summary of the grid search results, which is based on 49 pairs of ${\delta }_{BO}$ and ${\delta }_{HF/FO}$values.

**Table 1:** Results of the grid search to find optimal time windows (${\delta }_{BO}$ and ${\delta }_{HF/FO}$) for ARDS detection algorithm

Measure	Min	Max	95% Confidence Interval
Accuracy	78.0%,	92.0%	84.4%±1.3%
Precision	62.5%,	91.7%	84.5%±2.2%
Recall	77.6%	93.4%	84.3%±1.8%
${F}_{1}$	53.1%,	80.8%	64.6%±2.9%

### NLP algorithms

#### Detection of bilateral infiltrates (BI)

We trained a sentence-based classifier to detect the evidence of BI in chest radiograph reports. A report is classified as positive if it includes at least one positive sentence. Figure A3 in the Appendix shows the process of developing the training set, which included 2,376 sentences.

Two clinicians labeled all the sentences independently as “positive” (i.e., providing evidence of BI) or “negative.” “Positive” was defined as including mention of both right and left lungs involvement of the following: infiltrate, opacity, consolidation, airspace disease, aspiration, and pneumonia. Unilateral lung involvement, presence of bilateral pleural effusion, and consolidations attributed directly to atelectasis only were labeled negative. If the radiologist qualified an improvement or worsening of BI, the sentence was labeled as positive. Conversely, the sentence was labeled negative if the impression qualified interval resolution or recovery of BI.

The clinician’s agreement rate was 88.0%. Inconsistencies were resolved by deliberation with other clinicians in the group. Furthermore, the group consulted a diagnostic radiologist to provide insight into the decision-making. Finally, 938 positive sentences (positive rate = 39.5%) were identified in the training set. Figure A4 in the Appendix shows a summary of the training set. The data were divided into train and test sets using stratified sampling at a 75:25 ratio.

We built a classification pipeline with three main steps: text preparation, vectorization, and classification. For each step, we experimented with different parameter settings and used grid search with five-fold cross-validation to find the architecture that returns the maximum ${F}_{1}$ score for the positive class. Table A1 in the Appendix lists all pipeline parameters.

The text preparation step involves removing tags, punctuations (except for question marks), numbers, and multiple whitespaces, unifying all variations of common phrases into a single form (e.g., ‘*please’* and ‘*pls’*, ‘*pneumonia’* and ‘*pna’*, ‘*campared to’* and *‘in comparison with’*), and converting common multi-word phrases into unigrams (e.g., *‘pulmonary edema’* to’ *pulmonaryedema’*, *‘consistent with’* to ‘*consistentwith’*, and, ‘*final report*’ to *‘finalreport’*) and replacing the results of MIMIC’s named entities with a generic name (e.g., replacing [**Doctor Last Name 107**] with *LastName*). We varied two parameters in this step: using Standard English versus a customized list of stopwords and whether to apply stemming or not.

We tested two approaches for vectorization, bag of words (BoW) and word embedding. For BoW, we implemented the term frequency-inverse document frequency (TF-IDF) weighting scheme. We experimented with TF-IDF parameters listed in Table A. The word embedding was implemented using spaCy’s pre-trained word vectors. The vector representation of each sentence was obtained by averaging its token vectors. We used singular value decomposition (SVD) for dimensionality reduction and incorporated the number of dimensions as a parameter in the grid search.

We examined six learning models for classification and experimented with their hyperparameters, summarized in Table A1. It is worth noting that Stochastic Gradient Descent (SGD) is an optimization technique for the training of different linear classifiers rather than a learning model by itself. For instance, SGD with the hinge loss function is equivalent to a linear support vector machine (SVM).

#### Detection of heart failure/fluid overload (HF/FO)

Following a similar approach, we developed a pipeline to extract evidence of HF/FO in chest radiology and echo reports. Echo reports tend to have a different syntax and lexicon than radiology reports. Hence, we developed separate classifiers for radiology and echo reports. The keywords used to find the relevant sentences are *cardiac shock*, *cardiac arrest*, *cardiac failure*, *fluid overload*, *volume overload*, *heart failure*, *CHF*, *hydrostatic, cardiogenic, hypervolemia, systolic dysfunction, diastolic dysfunction, LVSD*, and *LVDD*. After reviewing an initial training set, we decided to include the sentences before and after the focal sentence to capture the context better. Thus, the HF/FO classifier’s input is a three-sentence document where the keywords occur in the middle unless the keyword is in the first or last sentence, resulting in a two-sentence document.

For the radiology reports classifier, a training set of 2,000 documents was randomly generated from the patients’ study cohort. To achieve a representative variety, we picked a maximum of two documents per patient. Two clinicians labeled the documents blindly. CHF positivity was defined by the phrase or combination of phrases that suggested a cardiogenic etiology or stated the presence of heart failure, congestive heart failure (CHF), edema, or fluid or effusions. Reports that did not comment on pulmonary parenchyma or collectively did not suggest a cardiogenic etiology responsible for pulmonary abnormalities were labeled negative. The disagreements, which had a rate of 6.2%, were settled by discussion and consensus. The final training set included 1,808 documents with 1,020 positive examples (positive rate = 56.4%). We stratified-split the data into train and test at an 80:20 ratio. Figure A5 in the Appendix summarizes the HF/FO classifier’s training set.

Following a similar procedure, we developed a training set for the echo reports classifier, which consisted of 1,048 random documents with 534 positive examples (positive rate = 50.1%).

#### Deployment of large language models (LLMs)

The study was initially designed before the rapid emergence of Large Language Models (LLMs). Nevertheless, we experimented with a few of these transformer-based models using the original training datasets, which were developed with conventional NLP models in mind. Specifically, we developed LLM pipelines for BI detection using quantized versions of Meta’s Llama 70B, Mistral 7B, and Nous Hermes 2 Mixtral 8 × 7B DPO. Due to its better performance on our training dataset, Llama 70B was ultimately selected as the teacher model.

To guide the analysis, we developed a structured prompt tailored to the specific diagnostic criteria for BI in the context of ARDS. The prompt is outlined in Figure A6 in the Appendix. The model was instructed to determine whether the evidence was positive or negative and to provide a definitive answer when uncertain. To enhance the performance of the model, we employed prompt optimization using Python’s DSPy library. A balanced subset of 500 labeled BI sentences was randomly selected from the BI training dataset, with 300 for training, 100 for validation, and 100 for testing.

We employed the Chain of Thought (CoT) method to determine the BI label for each example. Despite the prompt instructing the model to categorize the evidence as either positive or negative, the model’s answers varied (e.g., “neutral,” “answer: negative,” “leaning towards positive” or “the sentence offers negative evidence”). To address this, we established a mapping to categorize all responses as either positive or negative. For responses that fell outside this mapping, we used TypedChainOfThought to access the entire answer and predict the label. If the prediction remained ambiguous, the rationale generated in the previous step was used for the prediction. This procedure was repeated up to five times to get a definitive answer regarding the presence of BI evidence. With this setting, a clear answer was obtained in every example in the training set. Next. we configured the DSPy’s BootstrapFewShotWithRandomSearch method to optimize the prompt using in-context learning. The training set of 300 randomly selected examples was used for this purpose. Overall accuracy served as the metric for evaluating performance.

## Results

### Accuracy of the BI classifier

For the BI classifier described in Secction 2.3.1, the grid search within the pipeline parameters’ space returned TF-IDF vectorization and SGD with the modified Huber loss function as the optimal configuration. Details of the optimal setting are highlighted in Table A1. We replicated the optimal classification pipeline 30 times with different random seeds for test/train split and shuffled the training after each epoch. Table 2 shows the summary for different accuracy metrics. Accuracy is measured based on the 25% of the data set aside for testing in each replication. To further explore the textual features contributing to the classifier’s performance, refer to Table A2 in the Appendix, which lists the top 25 terms and phrases for the detection of BI.

**Table 2:** Accuracy of the BI classifier (based on 30 random test/train splits of 2,376 sentences)

Class	Measure	Min	Max	95% Confidence Interval
Overall	Accuracy	89.1%	94.1%	91.9% ± 0.5%
Negative	Precision	90.8%	97.6%	94.9% ± 0.5%
	Recall	88.3%	95.5%	91.6% ± 0.7%
	${F}_{1}$	91.0%	95.1%	93.2% ± 0.4%
Positive	Precision	84.2%	93.0%	87.8% ± 0.8%
	Recall	86.0%	96.6%	92.4% ± 0.8%
	${F}_{1}$	86.1%,	92.6%	90.0% ± 0.6%

### Accuracy of the HF/FO classifiers

Similar to the BI pipeline, we conducted an extensive grid search with five-fold cross-validation to find the optimal pipeline structure for the two HF/FO classifiers. Table 3 summarizes the test data accuracy levels obtained in 30 replications of the grid search. The feature importance analysis is presented in Table A2, which shows the top 25 *n*-grams that are most influential in detecting positive references to HF/FO.

**Table 3:** Accuracy of the HF/FO classifiers (based on 30 random test/train splits of 2,000 radiology and 1,048 echo documents

Data source	Class	Measure	Min	Max	95% Confidence Interval
Radiology reports	Overall	Accuracy	83.4%	88.7%	86.1%±0.5%
	Negative	Precision	80.5%	89.7%	85.1%±0.9%
		Recall	77.7%	88.3%	82.5%±0.9%
		${F}_{1}$	81.1%	86.6%	83.8%±0.6%
	Positive	Precision	84.3%	90.6%	86.8%±0.6%
		Recall	84.7%	92.5%	88.8%±0.8%
		${F}_{1}$	85.2%	90.2%	87.8%±0.5%
Echo reports	Overall	Accuracy	96.6%	99.6%	98.4%±0.3%
	Negative	Precision	96.1%	100.0%	98.7%±0.4%
		Recall	95.3%	100.0%	98.0%±0.4%
		${F}_{1}$	96.5%	99.6%	98.3%±0.3%
	Positive	Precision	95.7%	100.0%	98.1%±0.4%
		Recall	96.3%	100.0%	98.7%±0.4%
		${F}_{1}$	96.6%	99.6%	98.4%±0.3%

### Accuracy of the BI classifier developed using LLMs

Using the LLM as outlined in the previous section, our best result for BI detection on the test set achieved an accuracy of 77%, with a recall of 76%, precision of 77.6%, and ${F}_{1}$ score of 76.8%. These figures were lower than those obtained using the NLP method described in Table 2. This outcome was expected given the constraints imposed by the sentence-based structure of the training set. As mentioned earlier, we segmented the radiology reports into individual one-sentence documents to build the BI classifier. Such brief excerpts often lack the necessary context for an LLM to accurately assign the correct label.

### Accuracy of the ARDS detection algorithm

For ARDSFlag, which incorporates the NLP algorithms described above as components, Table 4 presents the confusion matrix based on the 300 cases in the test set. The overall accuracy of the algorithm is 89%. There were 71 true positive cases (defined based on the manual review by two groups of clinicians), 57 of which were detected by the algorithm (recall = 80.3%). The precision for the positive class is 75% leading to an ${F}_{1}$ score of 77.6%. There were 229 true negative cases, 210 of which were correctly classified (specificity = 91.7%).

**Table 4:** Confusion matrix for the test sets. The predicted label is shown in rows. Columns show the true label determined based on an independent review of two groups of clinicians (e.g., the algorithm generated 57 true positive and 14 false negative cases)

	True Label		
Predicted Label	Positive ARDS	Negative ARDS	Total
Positive ARDS	57	19	76
Negative ARDS	14	210	224
Total	71	229	300

Table 5 shows the algorithm performance compared to the two other methods used in the literature: automated implementation of the Berlin criteria developed by Serpa Neto et al. [18] and the use of International Classification of Diseases (ICD) codes to define ARDS [22–25]. We reproduced the Serpa Neto et al. [18]’s method using its detailed description in Le et al. [9] and confirmed the results match. For ICD-based algorithms, we included patients with respiratory failure as their primary or secondary diagnosis (ICD-9 codes 518.51, 518.52, 518.81, and 518.82) and experimented with the inclusion of mechanical ventilation procedure (ICD-9 codes 96.70, 96.71, 96.72) (similar to, e.g., Schwager et al. [25] and Eworuke et al. [22]), and exclusion of patients with a primary diagnosis of heart failure (ICD-9 codes 410, 411, 412, 414, 428) (similar to, e.g., Liu et al. [26] and TenHoor et al., 2001 [24]). The highest accuracy was achieved by incorporating the respiratory failure and heart failure codes and not including the ventilation procedure. Table 5 shows the outcome of this optimal ICD configuration. The results show that the algorithm outperforms other methods in all measures.

**Table 5:** Comparison of accuracy of different ARDS detection methods

Method	Accuracy	Specificity	Recall	Precision	${F}_{1}$-score
Proposed algorithm	89.0%	91.7%	80.3%	75.0%	77.6%
ICD-9	60.0%	68.1%	33.8%	24.7%	28.6%
Serpa Neto et al. [18]	73.5%	85.2%	36.1%	43.3%	39.4%

**Fig. 3:**
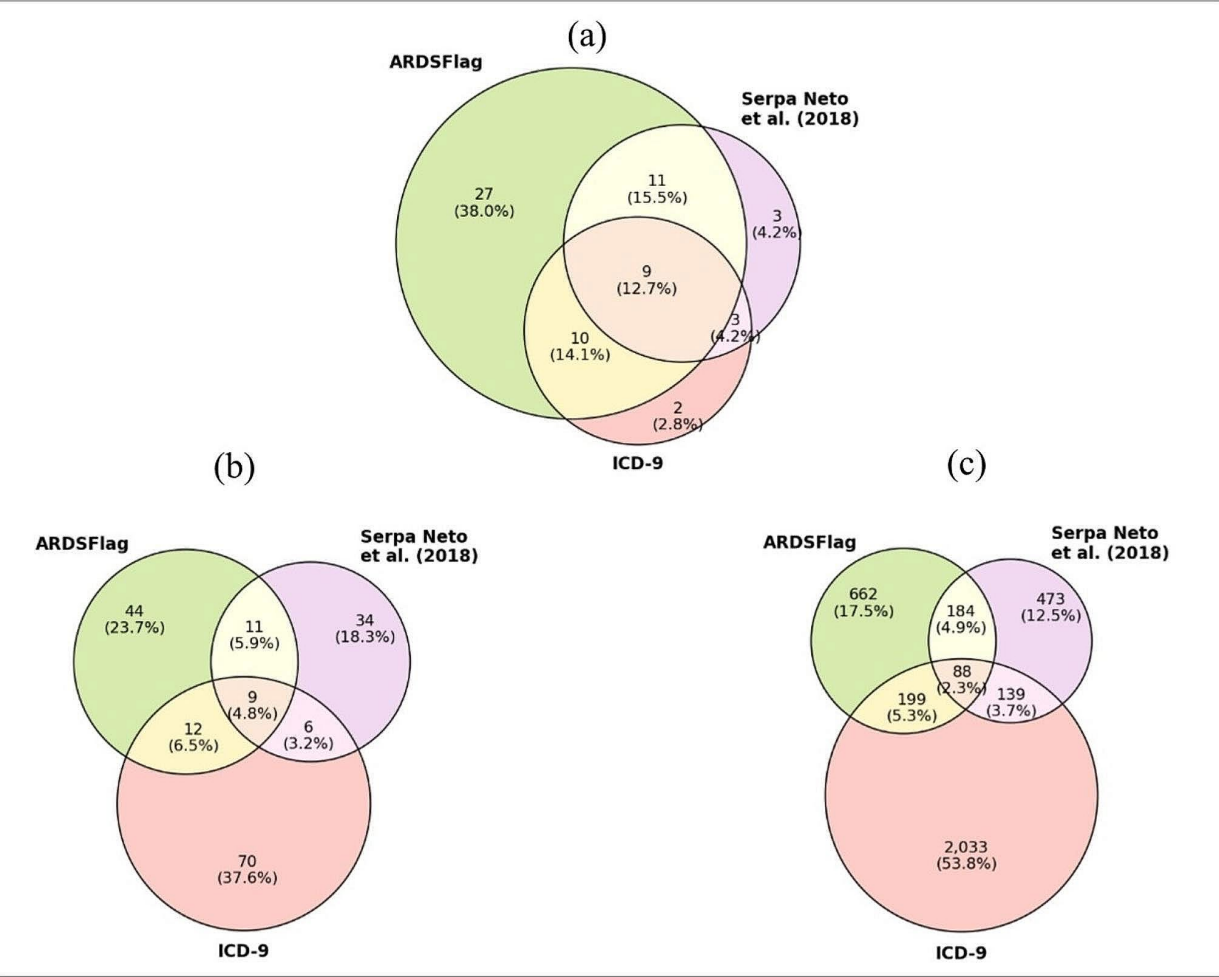
Venn diagram of positive ARDS cases detected by three methods within **(a)** the true positive cases in the test set (*n* = 71), **(b)** all test set cases (*n* = 300), and **(c)** the entire cohort (*n* = 19,534). The

The extent of overlap among the three methods is depicted using Venn diagrams in Fig. 3. Figure 3a shows their relationship over the 71 true positive cases in the test set. Our proposed algorithm (ARDSFlag) detected 57 (80.3%) ARDS cases, 27 (38.0%) of which are missed by both ICD-9 and Serpa Neto et al. [18] methods. However, ARDSFlag failed to detect 8 (11.3%) true positive cases that were identified by either one of the other two methods. All three methods failed to detect 6 (8.4%) true ARDS cases as defined by manual review by two groups of clinicians. Figure 3b shows the overlap among positive cases identified by the three methods within the 300 admissions in the test set, regardless of their true label. Collectively, the three methods detected 186 positive cases with an agreement rate of 4.8% (*n* = 9).

As evident from Fig. 3b, ICD-9 over-detects ARDS cases and Serpa Neto et al. [18] under-detects ARDS. We used the three methods to find ARDS cases in the entire study cohort and evaluate whether this pattern is generalizable. Figure 3c shows the results for the entire study cohort (*n* = 19,534). The ARDSFlag detected a total of 1,133 ARDS cases (prevalence of 5.8%), ICD-9 resulted in 2,459 (rate = 12.6%), and Serpa Neto et al. [18] generated 884 (rate = 4.5%). In line with the test set results, the three methods agree on only 2.3% (*n* = 88) of cases in the entire cohort, providing more evidence of wide discrepancy among different methods.

## Discussion

The literature has widely omitted HF/FO in identifying ARDS (e.g., Serpa Neto et al. [18]). Le et al. [9] refer to this departure from the Berlin criteria as one of the limitations of their study. They posit that it would be challenging to detect HF/FO using the available data without introducing bias. We performed a sensitivity analysis to estimate the effect of excluding HF/FO in ARDS detection by executing a version of the algorithm that does not include the criterion for the 300 cases in the test set. The second row in Table 6 shows the results. Evident from the results, failing to incorporate HF/FO results in a significant drop in the accuracy of the algorithm; overall accuracy decreases by 17.0%, precision by 24.4%, and F-score by 16.1%. Recall increases because the exclusion of HF/FO will produce more positive cases, leading to a lower miss rate.

The third and fourth rows in Table 6 provide details on the marginal effect of acuteness measures. We used two measures to evaluate the acute onset of ARDS. First, a tracheostomy procedure within seven days of admission violates acuteness. Without this criterion, two true negative cases would be misclassified as positive. Consequently, the marginal effect of using tracheostomy as a proxy for acuity on the model’s overall accuracy is 0.7%, and on recall is 1.1%. The second criterion was the time difference between onset and the earliest time when PEEP ≥ 5. As mentioned earlier, the algorithm requires this time difference to be less than seven days. By eliminating this criterion, the verdict changes for four cases; three true negative cases would move to the false positive set, and one false negative case would be resolved. Thus, this parameter has a net positive effect on the overall accuracy (89.0%-88.3%=0.7%) and the ${F}_{1}$ score (77.6%-76.8%=0.8%).

ARDSFlag requires a minimum of 48 h of mechanical ventilation unless the patient expires or opts for terminal elective extubation. By removing this condition, 12 true negative cases will be misclassified as positive, reducing the overall accuracy and ${F}_{1}$ score by, 89.0%-85.0%=4% and 77.6%-71.7% =5.9%, respectively.

Due to the architecture of ARDSFlag, a misclassification by the BI classifier does not always lead to an ARDS misclassification. For instance, missing the evidence of BI in one radiology report may be offset by finding the evidence in another report. In the manual review of all test set cases, we found two false negatives and three false positives that were caused by BI misclassification. As shown in the last row of Table 6, the BI classifier’s imperfection has led to a 1.7% drop in the overall accuracy and a 3.3% reduction in ${F}_{1}$ score.

**Table 6:** The effect of different components of ARDSFlag on accuracy. Arrow/values from the second row onwards show the direction/amount of change compared to the original version

Method	Accuracy		Specificity		Recall		Precision		${F}_{1}$-score	
ARDSFlag’s baseline accuracy		89.0%		91.7%		80.3%		75.0%		77.6%
ARDSFlag without the HF/FO classifier	**⇓**	-17.0%	**⇓**	-26.6%	**⇧**	14.1%	**⇓**	-29.4%	**⇓**	-16.1%
ARDSFlag without tracheostomy as a measure of acute onset	**⇓**	-0.7%	**⇓**	-0.9%		0.0%	**⇓**	-1.9%	**⇓**	-1.0%
ARDSFlag without the limit on the time of onset as a measure of acute onset	**⇓**	-0.7%	**⇓**	-1.3%	**⇧**	1.4%	**⇓**	-2.5%	**⇓**	-0.7%
ARDSFlag without requiring a minimum 48 h mechanical ventilation	**⇓**	-4.0%	**⇓**	-5.2%		0.0%	**⇓**	-10.2%	**⇓**	-5.9%
ARDSFlag with a hypothetically perfect classifier for BI	**⇧**	1.7%	**⇧**	1.3%	**⇧**	2.8%	**⇧**	3.7%	**⇧**	3.3%

**Fig. 4:**
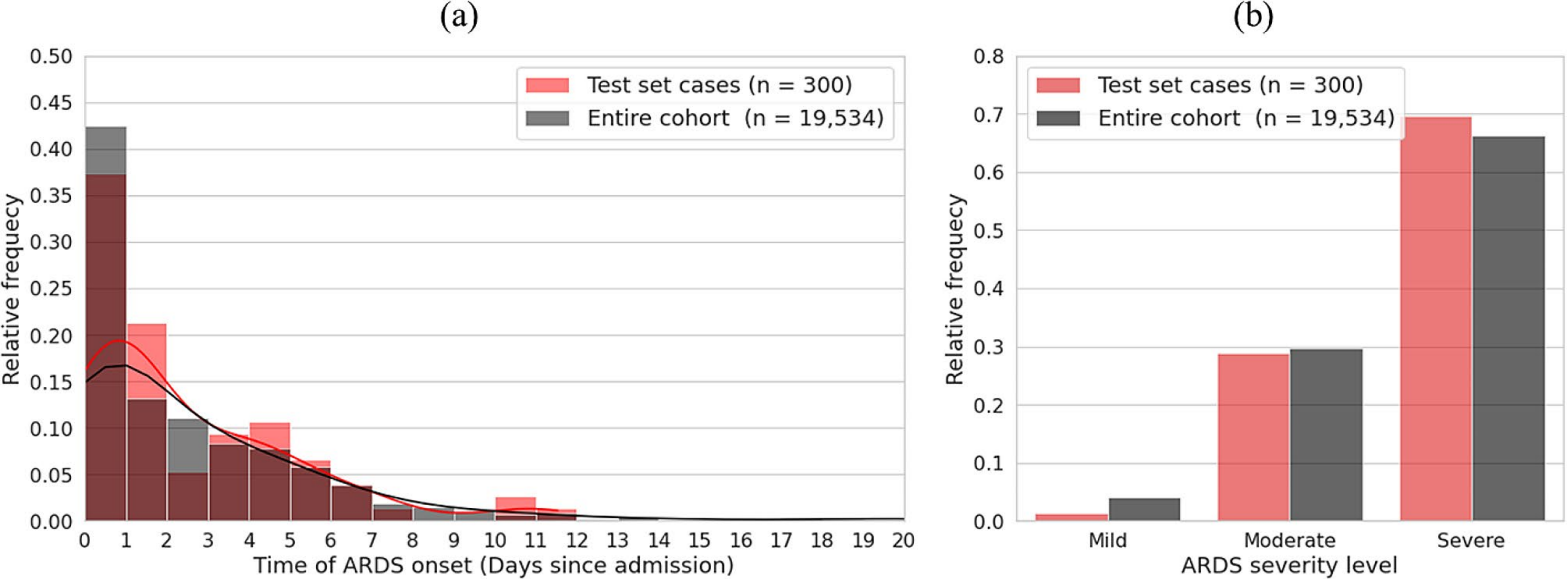
**(a)** Distribution of time of ARDS onset (days since admission) for the test set and the entire study cohort. **(b)** Distribution of ARDS severity in the test set and the entire study cohort

Further to the above sensitivity analysis, it is worthwhile to evaluate the distribution of time of ARDS onset and its severity. Figure 4 shows the two distributions for two patient populations: the test set ($n=300$) and the entire study cohort ($n=\text{19,534}$). As shown in the figure, most ARDS cases arise early in patients’ clinical course in both the test set and the cohort. This correlates clinically with the usual abrupt onset of ARDS following a precipitating cause.

Despite the overall accuracy and precision of the algorithm, there will be cases that go unidentified as ARDS. In one specific example reviewed, a patient with a history of pneumonectomy was deemed not to have ARDS by the algorithm. Even though the case was classified as ARDS based on clinician review, the algorithm would never identify it as such because the disease was technically unilateral. In addition, some cases showed initial evidence of cardiogenic edema. However, after aggressive diuresis, bilateral infiltrates remained present in repeat radiographic studies, and the patients were still profoundly hypoxemic. In these instances, the clinicians diagnosed the patients with ARDS. However, the algorithm would identify them as fluid overload cases and return a negative result. Regardless of the effectiveness of an algorithm, there will be nuances that will result in discrepancies between a calculated result and a clinician’s assessment.

## Limitations and future research

A notable limitation of this type of work is that we are emulating the Berlin criteria for ARDS. By design, this prevents the inclusion of rapidity of improvement and response to diuretics over time in someone who does not have preexisting heart failure. As such, if we retrospectively analyze some ARDS-positive cases, we see patients in whom the hypoxemia resolved after a few days of diuretic therapy. Therefore, in retrospect, these cases were most likely due to pulmonary edema and not ARDS. However, prospectively, this would not have been known. Perhaps including other relevant clinical data such as fever or leukocytosis in the criteria would help exclude such cases.

A general limitation of any algorithm is that it does not take into account nuanced details of a case that a clinician will be able to analyze. As stated, a patient who rapidly improves with diuretics would initially be classified as positive for ARDS without initial evidence of cardiac disease. This could also be true of renal patients who improve with hemodialysis. Another limitation is that the algorithm classifies bilateral infiltrates based on reports and not by actual image interpretation. There are sometimes cases where a report mentions bilateral disease, but on review of imaging, a clinician may determine that there is minimal bibasilar atelectasis. These nuances would only likely be identified by a clinician on review of a specific case, and as such is a limitation of the algorithm.

The current study utilizes the MIMIC-III dataset, which, while extensive, is confined to data from a single hospital and encompasses records until 2012. This limitation may raise questions about whether the algorithm can be effectively applied to more recent datasets or those from different healthcare systems. However, we expect the data preprocessing techniques, NLP algorithms, and the ARDS detection methodology to remain applicable across various settings. This expectation is founded on the standardized nature of clinical protocols and the consistent structure of medical language in radiology reports. To further validate these expectations, future research will focus on incorporating a broader array of data sources, thereby confirming the robustness of the proposed algorithm across various medical environments.

In this study, we introduced the ARDS graph, which consolidates data from various sources, including EHR, radiology, and ventilators. This graph provides access to essential information for the management of the ARDS. By integrating a real-time display of the ARDS graph into the clinical workflow, clinicians can rapidly access comprehensive data to make informed decisions, thereby potentially increasing both the efficiency of care and the quality of patient outcomes. Future initiatives can focus on deploying this system and conducting prospective studies to assess its impact in real-world settings.

We used LLMs to evaluate their performance in detecting positive references to BI. As noted in the Methods section, TF-IDF vectorization with SGD classification outperformed the LLMs. This finding confirms that more complex models do not inherently lead to better outcomes. The reason for LLMs’ worse performance may be attributed to the sentence-based structure of the proposed classifiers, which were developed without considering the use of LLMs. We believe such brief excerpts often lack the necessary context for an LLM to accurately assign the correct label. In future research, we plan to expand our training datasets to encompass entire radiology reports, which should better leverage the potential of LLMs.

### Electronic supplementary material

Below is the link to the electronic supplementary material.

Supplementary Material 1

Supplementary Material 2

Supplementary Material 3

## Data Availability

The 400 admissions that were manually labeled for ARDS are available in supplementary files along with ARDSFlag, Serpa Neto et al., and ICD results. The data supporting this study’s findings are available from the corresponding author upon reasonable request. The MIMIC-III database is publicly available (refer to https://mimic.mit.edu/docs/gettingstarted/ for instructions).
