# Supplementary material for: ARDSFlag: an NLP/machine learning algorithm to visualize and detect high-probability ARDS admissions independent of provider recognition and billing codes
Source: BMC Med Inform Decis Mak. 2024 Jul 16;24:195. doi: 10.1186/s12911-024-02573-5 (PMC11250933; doi:10.1186/s12911-024-02573-5)
Supplement: Supplementary file 1 — Supplementary Material 1 [file 12911_2024_2573_MOESM1_ESM.docx]

# Appendix

**Figure A1. ARDS graph for** **HADM-ID=117986.** The *ARDS graph* shows time series data on all the parameters relevant to ARDS detection, including P/F ratio, PEEP, bilateral infiltrates in the chest imaging reports, and heart failure/fluid overload in the chest imaging/echocardiogram/respiratory reports. The graph shows the earliest record of the tracheostomy procedure or oxygen delivery via tracheostomy ($T_{trach}^{0}$). Moreover, the graph shows the times of intubation, extubation, and discharge and the discharge disposition, which is “Facility” in this example.


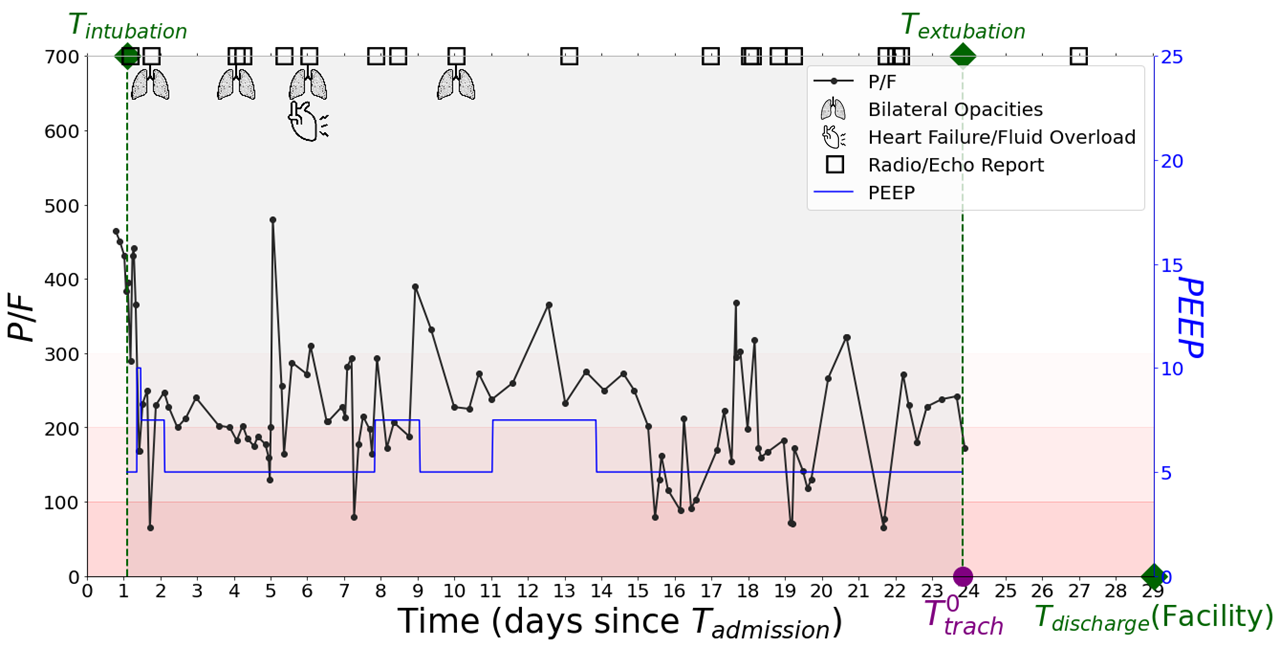


**Figure A2. Sample cases to demonstrate the ARDS detection algorithm:** **a.** HADM_ID=183612, Not ARDS because of cardiogenic cause. The short intubation length does not play a role because the patient expired within 48h of intubation. **b.** HADM_ID=105780, Not ARDS, because tracheostomy record (procedure or *O*_2_ delivery) upon admission violates the acute onset condition. **c.** HADM_ID=138174, Not ARDS because of cardiogenic cause. **d.** HADM_ID=115324, Not ARDS because the onset time (i.e., the first time P/F and PEEP condition is satisfied within bilateral infiltrates window, which is around Day 20) is beyond seven days after receiving PEEP≥5. **e.** HADM_ID=117986, Not ARDS because of cardiogenic cause; the first evidence of heart failure is detected around Day 6 (i.e., $T_{HF/FO}^{0}=6$), which renders heart failure as the origin of any edema observed from Day 1 onward ($T_{HF/FO}^{0}-\delta_{HF/FO}=6-5 )$. **f.** HADM_ID=126573, ARDS even though the length of intubation is less than 48 hours because the patient expires in that period.

**
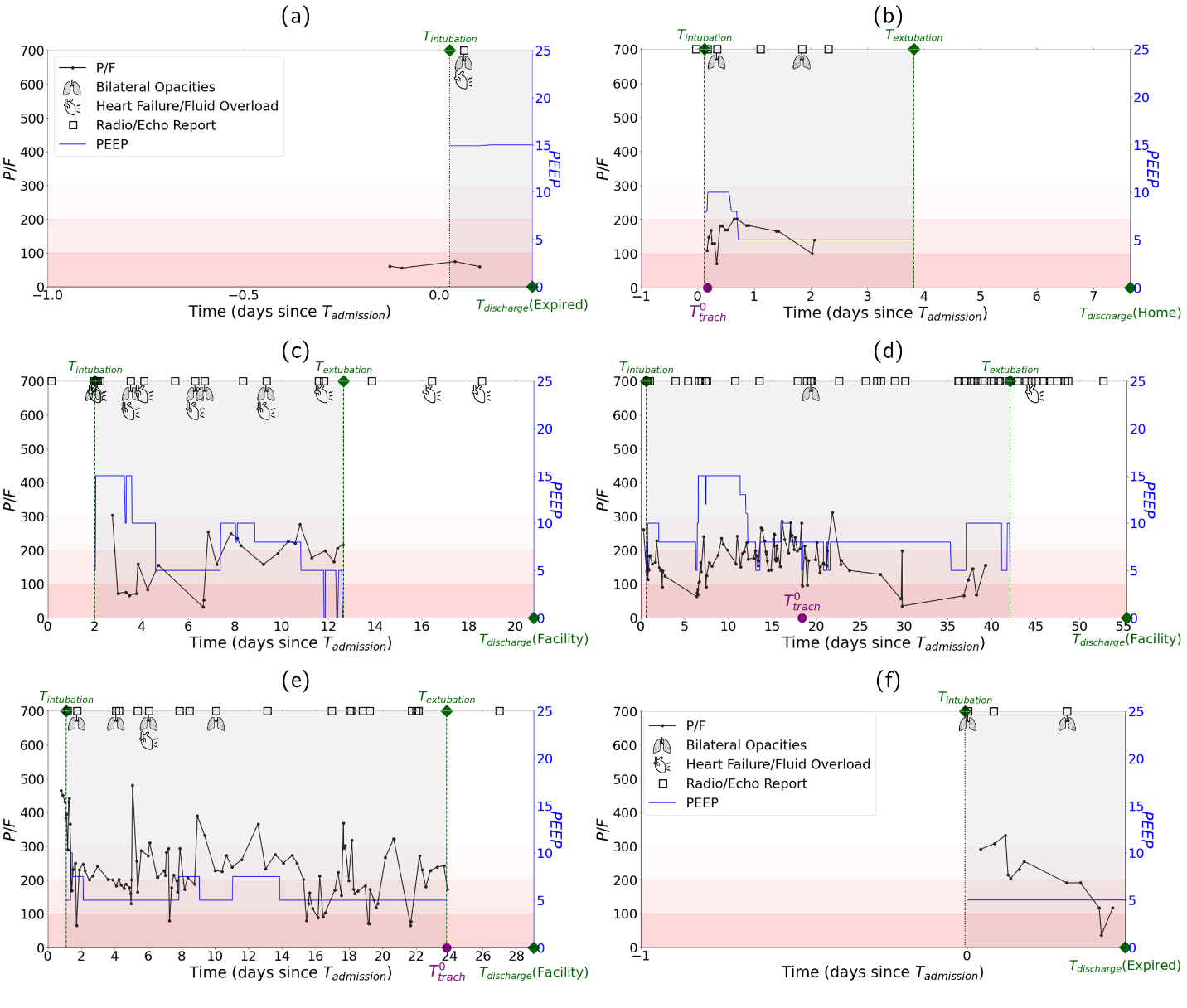
**

**Figure A3.** **The process of developing the training set for detection of bilateral infiltrates.** We used the following rule for sentence selection in Step 4: Use of any of the words in Set 1 (see below) or co-occurrence of at least one word from Set 2 and one from Set 3. Set 1 = {*opacity*, *infiltrate*, *consolidation*, *airspace disease*, *pneumonia*, *aspiration*, *ARDS*, *respiratory distress syndrome*}, Set 2 = {*bilateral, biapical, bibasilar, widespread, diffuse, perihilar, multifocal, extensive, both, lungs, left, right* }, and Set 3 = {*marking, infection, pattern, density, abnormality, haziness, hazy, process*}. Regular expressions were used to include keywords’ variations (e.g., *wide-spread*, *air-space* *disease,* and *opacities*). To create a more balanced dataset, we built an initial classifier based on 500 sentences labeled by one clinician and used it to generate 2,000 sentences that were more likely to be positive. The final training set included 2,376 sentences, 500 from Step 6 and 2,000 from Step 8, minus duplicates and non-sentences. Two clinicians labeled all sentences, resulting in 938 positive and 1,438 negative sentences.


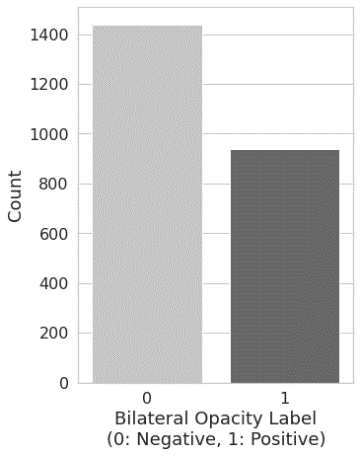

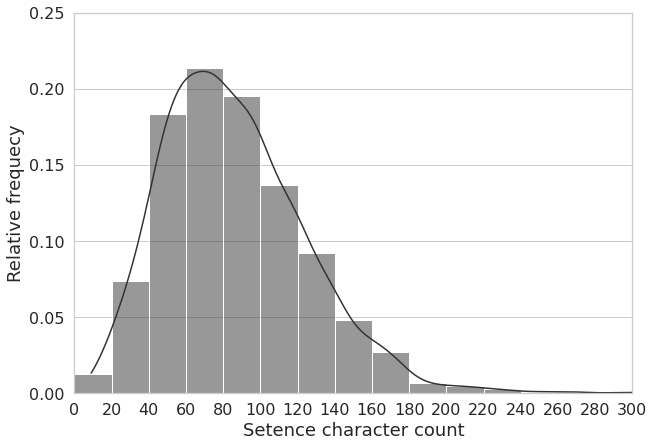

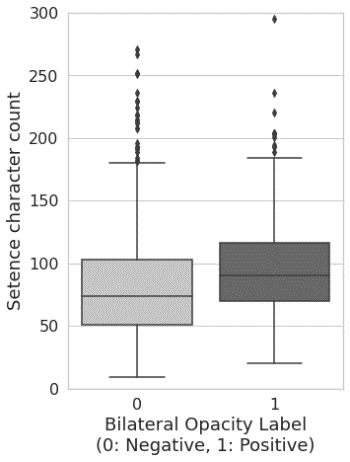


(a) (b) (c)


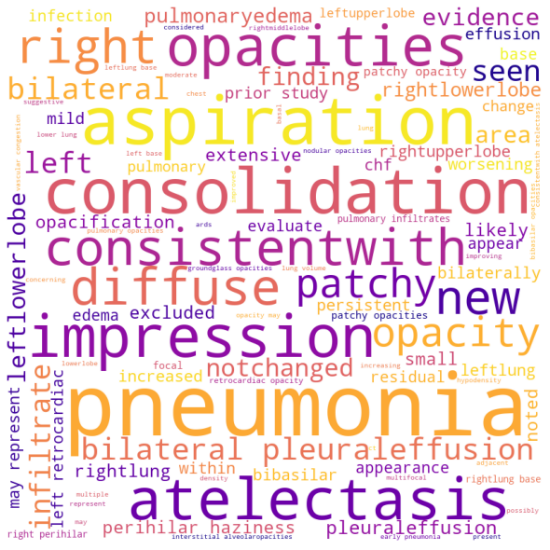

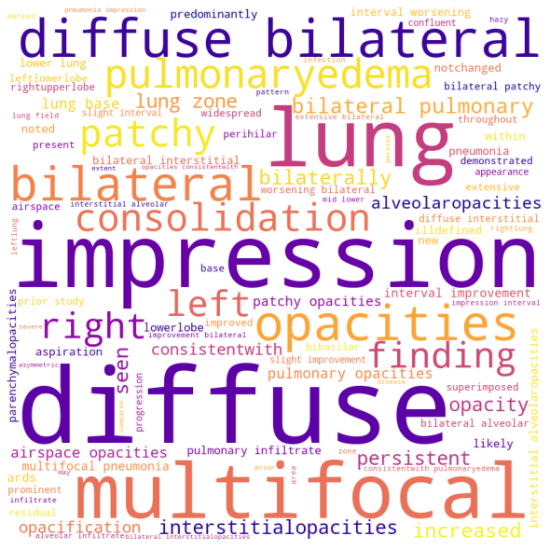


(d) (e)

**Figure A4. Describing the training set for bilateral infiltrates detection:** **a.** frequency of positive and negative examples ($n=2,376$); **b.** Histogram of the length of examples; **c.** distribution of length of examples by label; **d.** word cloud of unigrams and bigrams in negative examples; **e.** word cloud of unigrams and bigrams in positive examples.

Table A1. Classification pipeline hyperparameters for bilateral infiltrates detection (optimal values are boldfaced and underlined)

| Step | Approach | Parameters |
| --- | --- | --- |
| Text preparation | – | - Stop words = {Standard English, **Custom list of stopwords**} - Stemming = {on, **off**} |
| Vectorization | **TF-IDF** | - $n$-gram range = {only unigrams, **unigrams and bigrams**, unigrams and bigrams and trigrams} - Exclude terms that occur in over $m\%$ of documents: $m$ = {**30**, 50, 70, 90, 100} - Exclude terms that occur in less than $n\%$ of documents: $n$ = {**0**, 10} - Keep only top $x$features (ordered based on total frequency): $x$ = {3000, 4000, 5000, 6000, 7000, **∞** } - Use inverse-document-frequency reweighting: {**Yes**, No} |
|  | Word Embedding | - SVD dimensions: {50, 100} |
| Classification | **Stochastic gradient descent (SGD)** | - Loss function = {*Hinge, Log,* ***Modified Huber****, Squared Hinge, Perceptron*} - Regularization term = $\left\{ \boldsymbol{L}_{\boldsymbol{2}}, Elastic net \right\}$ - Maximum epochs = {50, 100, **200**} - Termination tolerance = {0.001, **0.0001**, 0.00001} - Regularization parameter ($\alpha$) = {**0.001**, 0.0001, 0.00001} |
|  | Support vector machine | - Kernel = $\left\{ Linear, Polynomial, RBF \right\}$ - Maximum solver iterations = {10, 50, 80} |
|  | Logistic regression | - Penalty = $\left\{ L_{2}, Elastic net \right\}$ - Maximum epochs = {10, 50, 80, 250} - Class weights = {*balanced*, {Negative:0.1, Positive:0.9}, {Negative:0.5, Positive:0.5}} |
|  | Multi-layer perceptron | - Hidden layer size = {1 , 2} - Number of neurons in the hidden layers = {(100), (50,50)} - Activation function = {*identity, logistic, tanh*} - Learning rate updates = {*constant*, *adaptive*}h, - Regularization parameter (α) = {0.00001, 0.000001} |
|  | Random forest | - Number of trees = {100, 150} - Maximum depth = {None, 10, 100, 1000} - Minimum samples required to split = {5, 10, 50} - Minimum samples required to be at a leaf node = {1, 3, 5} - Number of features to use to find the best split = {*auto*, *sqrt*, *log2*} |
|  | XGBoost | - Learning objective = {*logistic*, *hinge*} - Feature selector = {*shuffle*, *random*, *greedy*} |


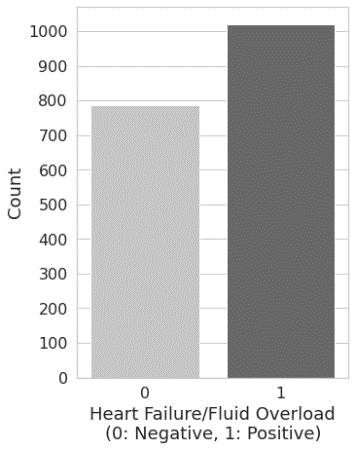

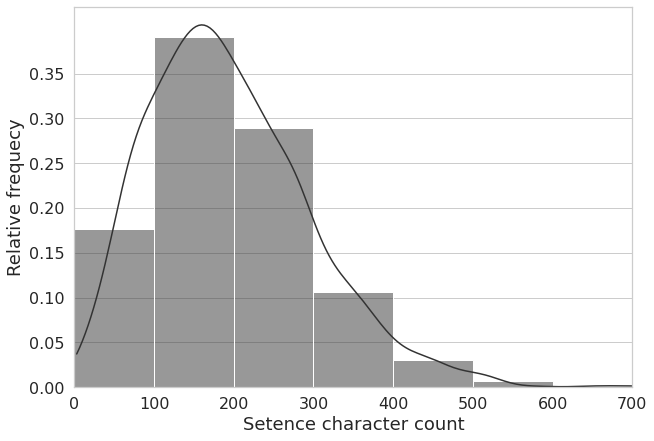

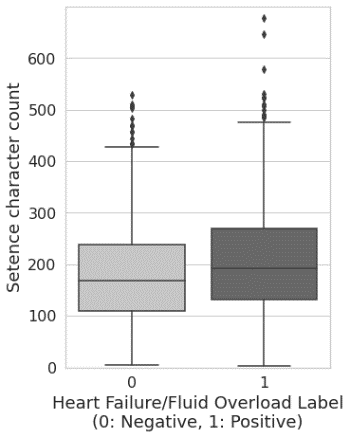


(a) (b) (c)


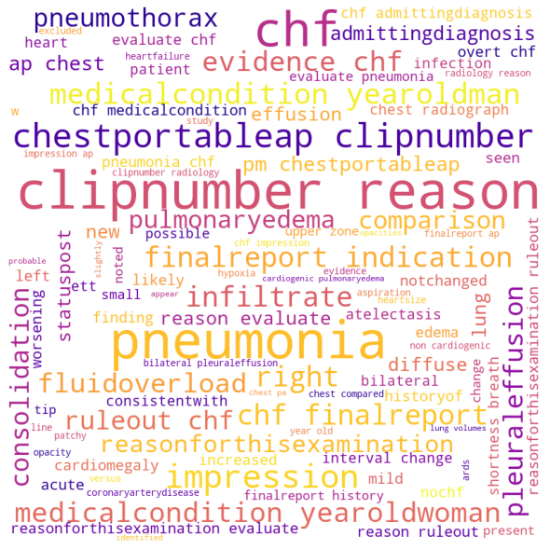

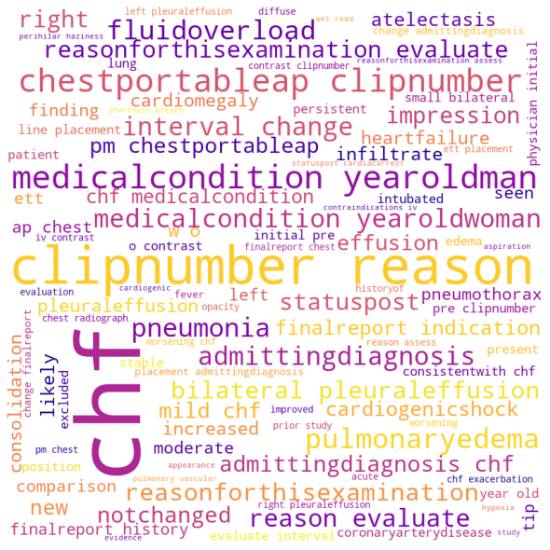


(d) (e)

**Figure A5.** **Describing the training set for heart failure/fluid overload detection:** **a.** frequency of positive and negative examples ($n=1,808)$; **b.** Histogram of the length of examples; **c.** distribution of length of examples by label; **d.** word cloud of unigrams and bigrams in negative examples; **e.** Word cloud of unigrams and bigrams in positive examples.

Table A2. Top 25 *n*-grams from Bilateral Infiltrates and Heart Failure/Fluid Overload text classifiers. This table lists the *n*-grams with the highest coefficients, indicating their importance in the classification model. Positive coefficients indicate phrases that positively correlate with the positive class, while negative coefficients suggest terms that negatively influence the class prediction.

|  | **Bilateral Infiltrates** | |  | **Heart Failure/Fluid Overload** | |
| --- | --- | --- | --- | --- | --- |
| **Rank** | ***n*-gram** | **Weight** |  | ***n*-gram** | **Weight** |
| 1 | bilateral | 3.30 |  | distribution | 3.30 |
| 2 | multifocal | 2.37 |  | clinical history | -1.91 |
| 3 | interstitial opacities | 2.06 |  | rales | 1.81 |
| 4 | atelectasis | -1.91 |  | ext | 1.68 |
| 5 | diffuse | 1.81 |  | cardiac mediastinal | 1.55 |
| 6 | lungs | 1.80 |  | indicate cardiac | -1.39 |
| 7 | both | 1.68 |  | evaluate | 1.32 |
| 8 | ards | 1.55 |  | entirely | -1.28 |
| 9 | cleared | -1.39 |  | acute chronic | 1.10 |
| 10 | rightlung | -1.38 |  | impression persistent | -1.09 |
| 11 | lung | 1.35 |  | admitting diagnosis respiratory | 1.01 |
| 12 | bilaterally | 1.32 |  | acute pulmonary edema | 0.99 |
| 13 | bilateral pleuraleffusions | -1.28 |  | ap portable | -0.94 |
| 14 | pulmonary edema | 1.28 |  | aneurysm medical condition | 0.94 |
| 15 | impression | 1.24 |  | venous line | 0.92 |
| 16 | pleuraleffusions | -1.24 |  | difficult | 0.89 |
| 17 | interstitial | 1.23 |  | displaced | 0.87 |
| 18 | no | -1.23 |  | mediastinal veins | -0.85 |
| 19 | pulmonary | 1.12 |  | doctor name | 0.81 |
| 20 | airspace | 1.10 |  | versus | 0.77 |
| 21 | chf | -1.09 |  | reason evidence | 0.76 |
| 22 | ill-defined | 1.03 |  | failure reason for this examination | 0.74 |
| 23 | widespread | 1.03 |  | no appreciable | 0.73 |
| 24 | alveolar | 1.01 |  | kidney | 0.72 |
| 25 | airspace opacities | 0.99 |  | cardiopulmonary process | 0.72 |

**Figure A6.** **Prompt used for LLM instructions in the detection of bilateral infiltrates in radiology report sentences.** This figure displays the prompt employed to direct the LLMs' analysis of radiology report sentences for identifying evidence of bilateral infiltrates.

| I have a sentence from a chest radiology report that I need to analyze to determine if it provides evidence of bilateral infiltrates (BI), which are significant for diagnosing acute respiratory distress syndrome (ARDS). Below are the specific criteria that define what constitutes evidence of BI. Please apply these criteria to the sentence provided and determine whether it indicates BI.  Criteria for BI Evidence Detection:  Positive Evidence: The sentence must mention the involvement of both the right and left lungs with any of the following terms: infiltrate, opacity, consolidation, airspace disease, aspiration, and pneumonia. The sentence is also considered positive if there is mention of improvement or worsening of BI.  Negative Evidence: The sentence mentions unilateral lung involvement, the presence of bilateral pleural effusion without explicit mention of bilateral lung involvement, consolidations attributed only to atelectasis, or an interval resolution or recovery of BI.  Based on the criteria provided, does this sentence offer positive or negative evidence of BI? If you suspect there is positive evidence but are not sure, answer: positive. If you cannot find positive evidence, answer: negative. Your answer must be either positive or negative. |
| --- |
